# Comparison of bactericidal and fungicidal efficacy of antiseptic formulations according to EN 13727 and EN 13624 standards

**DOI:** 10.3906/sag-1906-53

**Published:** 2019-10-24

**Authors:** Aslı ŞAHİNER, Ece HALAT, Evren ALĞIN YAPAR

**Affiliations:** 1 Department of Biology, Faculty of Science, Ege University, Bornova, İzmir Turkey; 2 Department of Analysis and Control Laboratories, Turkish Medicines and Medical Devices Agency , Sıhhiye, Ankara Turkey

**Keywords:** Antiseptic solutions, microbial efficacy, EN 13727, EN 13624, medical use

## Abstract

**Background/aim:**

In this study, the antibacterial and antifungal properties of the five most commonly used antiseptic formulations were evaluated in terms of different contact times and organic conditions.

**Materials and methods:**

Solutions of chlorhexidine digluconate, povidone iodine, isopropyl alcohol, hydrogen peroxide, and tincture of iodine were prepared and tested according to European standards EN 13727 and EN 13624 with different parameters.

**Results:**

The results showed that isopropyl alcohol (70% v/v) and tincture of iodine (2%) had greater bactericidal and fungicidal activity against the four tested bacteria and two fungi in all conditions.

**Conclusion:**

When the results of the five different active substances were quantitatively evaluated regarding their bactericidal and fungicidal activities, it was found that contact time and organic load significantly affected the antiseptic efficacy.

## 1. Introduction

Antiseptics are defined as substances or preparations that enable the treatment of living tissues by killing or inhibiting microorganisms in order to prevent or limit the risk of infection. In order to carry out their purpose, these products include active substances such as quaternary ammonia, chlorhexidine, alcohols, oxidants, and organic acids. The purpose of these substances are to control the skin and mucocutaneous microbial colonization on skin and wound surfaces [1]. Five antiseptic formulations that are commonly used for antisepsis are chlorhexidine digluconate (CHX) solution, povidone-iodine (PVP-I) solution, isopropyl alcohol (IPA) (70% v/v), hydrogen peroxide (HP) (3%) solution, and tincture of iodine (TI) (2%) solution. As for the areas of use, CHX solution (2%) is an antiseptic formulation that can be used for skin and hand disinfection. PVP-I is a chemical complex of polyvinylpyrrolidone and elemental iodine used as a disinfectant in various pharmaceutical formulations, whereas IPA can be used for hand and equipment disinfection. HP is an antiseptic that can be used to prevent infections of minor cuts, scrapes, burns of skin, sores, and gingivitis of the oral cavity, and tincture of iodine solution, also known as weak iodine solution, is an antiseptic used for preoperative skin preparation of patients and helps to reduce bacteria that can potentially cause skin infections [1,2]. In this study, the antibacterial and antifungal properties of the above-mentioned five most commonly used antiseptic solutions were evaluated in terms of different contact times and organic conditions. In this context, it was aimed to prepare solutions of CHX, PVP-I, IPA, HP, and TI and to compare their bactericidal and fungicidal activity according to European standards EN 13727 [3] and EN 13624 [4]. Four bacterial and two fungal strains were used to assess the effectiveness of each antiseptic formulation. These test strains were *Escherichia coli *K12,* Pseudomonas aeruginosa*,* Staphylococcus aureus*,* Enterococcus hirae*,* Candida albicans*, and *Aspergillus brasiliensis *(formerly known as* Aspergillus niger*).

## 2. Materials and methods

### 2.1. Media and chemicals

Chlorhexidine digluconate (20%,) polyvinylpyrrolidone (PVP)–iodine complex, sodium phosphate dibasic dehydrate, citric acid, isopropyl, polysorbate 80, catalase, hydrogen peroxide, bovine serum albumin, and iodine were obtained from Sigma Aldrich (USA). Malt extract agar (MEA) and tryptic soy agar (TSA) were purchased from Oxoid (UK). Maximum recovery diluent, lecithin, and defibrinated sheep blood were obtained from Merck, Alfa Aesar, and Thermo Fisher Scientific, respectively.

### 2.2. Preparation of antiseptic solutions

Five solutions, namely 2% chlorhexidine digluconate solution (CHX 2%), 7.5% povidone-iodine solution (7.5% PVP-I), 70% isopropyl alcohol (70% IPA), 3% hydrogen peroxide (3% H2O2), and 2% tincture of iodine (2% TI) were used as antiseptic solutions. Each solution was formulated as follows: 

- Formulation A: CHX 2% was prepared by mixing 100 mL of 20% chlorhexidine digluconate with 900 mL of distilled water.

- Formulation B: 7.5% PVP-I was prepared by adding 7.5 g of PVP-iodine 30/06 to 80 mL of citric acid-phosphate buffer solution (pH 5.0). The mixture was homogenized by a magnetic mixer (Hanna, Italy) at room temperature for 5 min. After that, the volume of solution was completed to 100 mL with citric acid-phosphate buffer solution.

- Formulation C: 70% IPA was obtained by mixing 700 mL of isopropyl alcohol with 300 mL of distilled water. 

- Formulation D: 3% H2O2 was prepared by adding 10 mL of 30% hydrogen peroxide to 90 mL of distilled water.

- Formulation E: 2% TI was prepared by mixing 2% iodine and 2.5% potassium iodide in 50 mL of 90% ethanol. The volume of the mixture was completed to 100 mL of with distilled water. 

### 2.3. Neutralizers and interfering substance

In order to limit the contact time of the antiseptics, the active substances constituting the antiseptic solutions were neutralized by specific substances. Neutralizer compositions were prepared according to EN 13727 and 13624 standards [3,4] as shown in Table 1.

**Table 1:** Neutralizer compositions.

Active substance	Neutralizer
Hydrogen peroxide	Polysorbate 80, 50 g/L; lecithin, 10 g/L; catalase 0,25 g/L
Iodine	Sodium thiosulfate, 15 g/L; polysorbate 80, 30 g/L; lecithin, 3 g/L
Alcohol	Saponin, 30 g/L; polysorbate 80, 30 g/L; lecithin, 3 g/L
Chlorhexidine digluconate	Saponin, 30 g/L; polysorbate 80, 30 g/L; lecithin, 3 g/L; L-histidine, 1 g/L

Organic load is an important factor reducing the effectiveness of disinfectants. Therefore, according to the application area of the disinfectant, bovine serum albumin (BSA) and defibrinated sheep blood were used as interfering agents. The dirty condition was established with a mixture of 3.0 g/L BSA and 0.3% defibrinated sheep blood, while 0.3 g/L BSA was employed for the clean condition.

### 2.4. Microorganisms and growth conditions

The antimicrobial effects of the antiseptic solutions were evaluated on four bacterial strains and two fungi. The bactericidal tests were performed with *Staphylococcus aureus* ATCC 6538, *Escherichia coli* K12 NCTC 10538, *Pseudomonas aeruginosa* ATCC 15442, and *Enterococcus hirae* ATCC 10541. *Candida albicans* ATCC 10231 and *Aspergillus brasiliensis* ATCC 16404 were used for the fungicidal tests.

Before the antimicrobial tests, microorganisms were grown on specific media. All strains of bacteria from stock cultures were incubated on tryptic soy agar (TSA) at 37 °C for 24 h. After that, the resulting colonies were inoculated again on TSA in the same conditions. *Candida albicans* ATCC 10231 was grown on malt extract agar (MEA) as mentioned above. *Aspergillus* *brasiliensis* ATCC 16404 suspension was prepared with the resuspension of lyophilized Bioball (BioMérieux, France). 

### 2.5. Antimicrobial testing

Bactericidal and fungicidal efficacy tests were performed according to EN 13727 and EN 13624, respectively [3,4]. The antimicrobial tests were carried out at 20 °C using a water bath (Nüve, Turkey). Concentrations of bacterial and yeast test suspensions were adjusted to 1.0 McFarland standard with a densitometer (Biosan, Latvia). Lyophilized culture Bioball (Biomerioux) was used for *Aspergillus brasiliensis* spore suspension. One milliliter of each microorganism suspension was mixed with an equivalent volume of interfering substance in sterile tubes for 2 min. Afterwards, 8 mL of disinfectant were added to tubes without mixing. The tubes were then kept at 20 °C for 1 and 5 min. At the end of contact time, 1 mL of the mixture was transferred to a new tube containing 8 mL of neutralizer and 1 mL of sterile distilled water. The tubes were mixed by vortex for 10 s. After the neutralization process, the living microorganisms were enumerated by the pour plate technique. Inoculated petri dishes were incubated at 37 °C for bacteria and 30 °C for fungi for 48 h. Calculations were made by subtraction of logarithmic values of control and test results. The efficacy limit of antiseptics is 4 log for fungi and 5 log for bacteria according to the EN 13624 and 13727 standards, respectively. All studies were performed in duplicate.

## 3. Results

The antimicrobial activity of the five antiseptic solutions in terms of their active substances tested under different contact times and organic conditions are given in Table 2. Toxic effects of neutralizers and interfering substances and effectiveness of the neutralization process have also been validated according to the EN 13727 and EN 13624 standards [3,4].

**Table 2:** Bactericidal and fungicidal activity of tested formulations.

Logarithmic reduction of microorganisms after contact time											
Test organisms	Formulations	A (CHX 2%)		B(PVP-I 7.5%)		C (IPA 70%)		D (HP 3%)		E (TI 2%)		Interfering substance	1 min	5 min	1 min	5 min	1 min	5 min	1 min	5 min	1 min	5 min
	S. aureus	Clean condition	4.95	>5.05	3.24	>5.05	>5.36	>5.36	1.32	3.38	>5.52	>5.52	Dirty condition	4.84	>5.05	2.52	>5.05	>5.36	>5.36	0.78	0.90	>5.52	>5.52
E. coli K12		Clean condition	>5.52	>5.52	>5.52	>5.52	>5.37	>5.37	2.80	2.64	>5.17	>5.17	Dirty condition	4.94	>5.52	>5.52	>5.52	>5.37	>5.37	2.12	2.96	>5.17	>5.17
	P. aeruginosa	Clean condition	4.38	>5.38	>5.03	>5.38	>5.08	>5.08	3.66	5.22	>5.37	>5.37	Dirty condition	4.12	>5.38	>5.03	>5.38	>5.08	>5.08	3.30	5.08	>5.37	>5.37
E. hirae		Clean condition	>5.21	>5.21	3.55	>5.05	>5.46	>5.46	0.18	0.16	>5.05	>5.05	Dirty condition	>4.04	>5.21	2.78	>5.05	>5.45	>5.46	0.11	2.25	>5.05	>5.05
	C.albicans	Clean condition	3.52	>4.52	>4.04	>4.52	>4.26	>4.26	0.11	0.21	>4.21	>4.21	Dirty condition	3.27	>4.52	3.04	>4.52	>4.26	>4.26	0.05	0.09	>4.21	>4.21
A. brasiliensis		Clean condition	1.76	1.98	2.24	2.69	>4.33	>4.33	0.07	0.11	>4.05	>4.05	Dirty condition	0.88	1.76	1.25	2.53	>4.33	>4.33	0.04	0.04	>4.05	>4.05

The results of the antimicrobial efficacy tests were evaluated according to the logarithmic limits given in the standards. Formulations not showing 5 log reduction for bacteria and 4 log reduction for fungi were considered to be ineffective. The results showed that Formulation D (HP 3% v/v) had no bactericidal and fungicidal activity in the defined conditions, especially in the dirty condition, and the microbial activity of this antiseptic solution was determined to be very low. Formulation C (IPA 70% v/v) and Formulation E (tincture of iodine 2%) had greater bactericidal and fungicidal activity against the four tested bacteria and two fungi in all conditions. Formulation A (CHX 2%) and Formulation B (PVP-I 7.5%) had no fungicidal activity against *A. brasiliensis *in both dirty and clean conditions. 

## 4. Discussion

Antiseptic solutions, with different biocidal agents that are used for hand disinfection, mucous membranes, and wound surfaces, are used to reduce the risk of bacterial contamination in medical areas and to prevent cutaneous and mucocutaneous infections. Although an antiseptic solution has high antimicrobial effects, it should not be an irritant due to its use on skin and on wound surfaces [5]. This limits the types of active substances that can be used in antiseptic formulations. The antiseptic solutions used in the medical field generally include one of the following active substances: CHX, alcohol, benzalkonium chloride, iodine solutions, hydrogen peroxide, or any suitable combinations thereof. The effect mechanisms of active substances used as antimicrobial agents against microorganisms show variations. Some active substances disrupt the integrity of the cell wall or cell membrane, inhibiting the intracellular transfer of substances, while some of them degrade the enzymes and some inhibit the transcription and translation mechanisms by disrupting the structure of the DNA or RNA. In this study, five different formulations were prepared from the most commonly used active agents in commercial antiseptic solutions. Bactericidal and fungicidal activities of these formulations were compared by using the phase 2 step 1 in vitro test methods EN 13727 [3] and EN 13624 [4]. The purpose of these studies was to determine the bactericidal and fungicidal activity of the disinfectant and antiseptic solutions under practical conditions with respect to their intended use. The experiment was carried out using different times and interfering substance conditions. Thus, the effects of contact time and organic challenge on the microbial activity of the active substances were also observed. Formulation C demonstrated homogeneous results regardless of the time and interfering substance conditions. It provided the desired logarithmic reduction in all conditions on the four bacterial and two fungal strains tested. IPA shows fast and broad-spectrum antimicrobial activity against vegetative bacteria (including mycobacteria), some viruses, and fungi. Although it is known to inhibit sporulation and spore germination, it is not sporicidal. The antimicrobial activity of IPA is quite low at concentrations below 50%. The specific mode of action of IPA is to cause membrane damage and rapid denaturation of proteins [2]. CHX showed lower microbial activity in the dirty condition where the organic load was high, and it had no fungicidal activity against spores of *A. brasiliensis* mold in any tested conditions. CHX is the most widely used biocidal agent in antiseptic solutions, in particular in handwashing and oral solutions, due to its good bactericidal efficacy and low irritation. The intake of CHX to bacteria and yeast cells has been shown to be extremely rapid. CHX produces damage to the outer cell layers but is insufficient to induce lysis or cell death. The substance then attacks the bacterial cytoplasmic or inner membrane or the yeast plasma membrane. CHX in high concentrations causes coagulation of components within the cell. It is a disadvantage that CHX activity is pH-dependent and decreases in the presence of organic compounds [6]. The PVP-I results were similar to those of CHX, but the contact time greatly influenced the efficacy of this formulation. Increased contact time also increased PVP-I efficiency. PVP-I is a complex of iodine and polyvinylpyrrolidone. Povidone is a polymer that does not have antimicrobial activity, but it allows the transport of iodine from cell membranes. After iodine passes through the cell walls of microorganisms, it forms complexes with amino acids and unsaturated fatty acids, resulting in inhibition of protein synthesis and degradation of the cell membrane. The antimicrobial efficacy of PVP-I is influenced by temperature, contact time, the presence and type of organic and inorganic compounds, and pH [7]. Formulation D did not achieve the desired logarithmic reduction in the standards under the conditions tested. It has been observed that the efficacy on catalase-positive organisms is very low due to the breakdown of hydrogen peroxide to water and oxygen. Hydrogen peroxide is a commonly used active substance for disinfection and antisepsis since it is a colorless and clear liquid. H2O2 acts as an oxidant, producing hydroxyl free radicals that inhibit essential cell components such as lipids, proteins, and DNA. The presence of catalase or peroxidases at low concentrations increases the tolerance of organisms. Therefore, higher concentrations of H2O2 and longer contact times are required to achieve the desired antimicrobial activity. The rapid reduction in the presence of high organic compounds is the disadvantage of this antiseptic [2]. Due to its ethanol content, tincture of iodine displayed very high antimicrobial activity against microorganisms including *Aspergillus* spores. Although it is a highly effective antiseptic, its use is limited because of its irritant properties. 

The combination of contact time and concentration of active substance and organic load in the environment play an important role in disinfectant efficacy. Healthy skin and wound mucosa have significant protein loads, which causes partial inactivation of antiseptic solutions. Especially in infected wounds, antiseptics that do not show sufficient activity may cause some bacteria to develop resistance. As a result of antiseptic resistance, bacteria may also gain cross-resistance to some antibiotics.

In conclusion, when the results of five different active substances were quantitatively evaluated regarding their bactericidal and fungicidal activities, it was found that IPA and tincture of iodine were the most effective and hydrogen peroxide was the least effective. This study demonstrates that contact time and organic load significantly affect antiseptic efficacy.
